# IMPACT OF ENVIRONMENTAL CHARACTERISTICS ON SELF-RATED HEALTH AMONG THE VERY OLD IN GERMANY DURING THE COVID-19 PANDEMIC

**DOI:** 10.1093/geroni/igad104.1866

**Published:** 2023-12-21

**Authors:** Jaroslava Zimmermann

**Affiliations:** University of Cologne, Cologne, Nordrhein-Westfalen, Germany

## Abstract

Current research demonstrates negative impact of COVID-19 pandemic on health. Nevertheless, there is evidence that older population might be more resilient against the effect of pandemic than younger adults. Since very old adults are often underrepresented or excluded from these studies, the aim of this paper was to examine the link between environmental characteristics and self-reported health in very old population in Germany during COVID-19 pandemic. Data from the representative survey “Old Age in Germany” of the population aged 80 and older were used. The study sample included 10,578 community-dwelling as well as institutionalized persons. Multilevel analysis was conducted to investigate the association of environment characteristics on individual, and community level with self-rated health. The results showed that higher perceived walkability of outdoor environment, better housing condition, being closer attached to home environment, higher neighborhood trust, and living in a house increased self-rated health (individual level). On community level, residency in one of the communities in western Germany (compared to eastern Germany) was associated with better self-rated health. 35% of the variance in self-rated health was explained at individual and 21% at community level. The findings revealed that very old adults who lived in better home environment experienced better health during the COVID-19 pandemic in Germany. These findings may indicate that the capacity of the very old to adapt to health losses depends on available environmental resources. Therefore, improvements of home environment inside and outside of houses and apartments (e.g., ensuring barrier-free accessibility) may strengthen the resilience in later life.

